# Survival of Dental Implants Placed in Iliac, Fibula, and Radial Forearm Flaps: A Comprehensive Review

**DOI:** 10.7759/cureus.48031

**Published:** 2023-10-31

**Authors:** Bandar K Alzahrani, Bader Fatani, Hissah S Alshalawi, Rana M Almutairi, Hesham S AlRfydan, Maryam M Alhindi

**Affiliations:** 1 Dentistry, Prince Sattam bin Abdulaziz University, Alkharj, SAU; 2 Dentistry, King Saud University, Riyadh, SAU; 3 Dentistry, King Saud Bin Abdulaziz University for Health Sciences, Riyadh, SAU; 4 Oral and Maxillofacial Surgery, Ministry of Health - Health Qassim Cluster, Qassim, SAU; 5 Oral and Maxillofacial Surgery, King Saud University, Riyadh, SAU

**Keywords:** free flap, iliac flap, radial flap, fibula flap, dental implant

## Abstract

Various donor sites have been extensively documented in the literature for bone free flaps in head and neck reconstruction. These include the radius, scapula, rib, ilium, femur, fibula, and metatarsal bone. Among them, the fibula, ilium, and scapula are the most commonly used and studied for placing endosseous implants and for rehabilitation purposes. Each donor site has its own advantages and disadvantages, which depend on factors such as whether the reconstruction is for the maxilla or mandible, the required volume and length of the bone and soft tissue, and the location, extent, and type of defect that needs to be reconstructed. The aim of this current review is to comprehensively assess the existing literature on the survival of implants in fibula, radial, and iliac flaps.

## Introduction and background

Mandibular reconstruction presents a challenge for most maxillofacial surgeons due to two main factors [1]. Firstly, the region's anatomical diversity and the complex movements of the mandible, which are responsible for crucial oral functions such as chewing, swallowing, speech, and facial expressions, with significant impact on quality of life and social inclusion. Secondly, there is a high prevalence of oral cancer, which further affects the situation [1]. Mandibular reconstruction is often necessary to restore bone integrity following trauma, infection, large jaw cysts, ablative oral cancer surgery, or even prolonged use of removable dental prostheses that lead to bone resorption [1]. It is important to note that apart from mandibular reconstruction, other factors such as tumor size and location, involvement of the tongue, soft tissues, and remaining teeth also play a significant role in restoring oral functions [1].

Each of the donor sites has its advantages and disadvantages, which depend on factors such as whether reconstruction is for the maxilla or mandible, the required volume and length of the bone and soft tissue, and the location, extent, and type of defect to be reconstructed [2]. The donor sites carry varying levels of morbidity, and the flaps possess different characteristics based on factors such as the quantity, quality, and length of available bone, the possibility of performing osteotomy, the length of the vascular pedicle, the availability of the donor site, and the nature of the soft tissue skin paddle [2].

Mandibular reconstruction is a complex and challenging procedure in reconstructive craniomaxillofacial plastic surgery. It is performed to address a range of conditions, including cancer resections, traumatic injuries, and osteoradionecrosis [3]. The mandible plays vital roles in functions such as appearance, mastication, speech, and oral competence, and the goal of reconstruction is to restore both form and function [3]. Different surgical techniques, such as non-vascularized and vascularized grafts, can be used to achieve this. Non-vascularized bone grafts (NVBGs) are suitable for specific situations such as delayed reconstructions of small traumatic defects. However, they have limitations due to the lack of blood supply, resulting in slow healing, higher risks of infection, non-union, and fractures. They are also prone to osteoradionecrosis when combined with radiation therapy [3].

The introduction of microvascular surgery has transformed mandibular reconstruction significantly, particularly after radiation treatment for cancer. Vascularized bone grafts (VBGs) have their own blood supply, which shortens the time required for union [3]. Free VBGs, especially those using fibula grafts, have shown significantly better outcomes compared to non-vascularized options such as reconstruction plates and bone grafts, particularly for defects longer than 6 cm and involving the parasymphyseal and/or anterior border regions. Comparisons directly made between NVBGs and vascularized bone flaps (VBFs) have shown that VBFs are superior in terms of bony healing, functional outcomes, and aesthetic scores related to aspects such as diet, speech, and symmetry along the middle line [3]. VBGs outperform NVBGs significantly in cases of mandibular defects larger than 6 cm or involving previously radiated tissue. Among the options for VBGs, the fibula, radial forearm, scapula, and iliac crest are available. The fibula flap is the most commonly chosen option for the reconstruction of the mandible [3]. This review aims to discuss the survival of dental implants placed in fibula, radial, and iliac flaps.

## Review

Methods 

**Figure 1:**
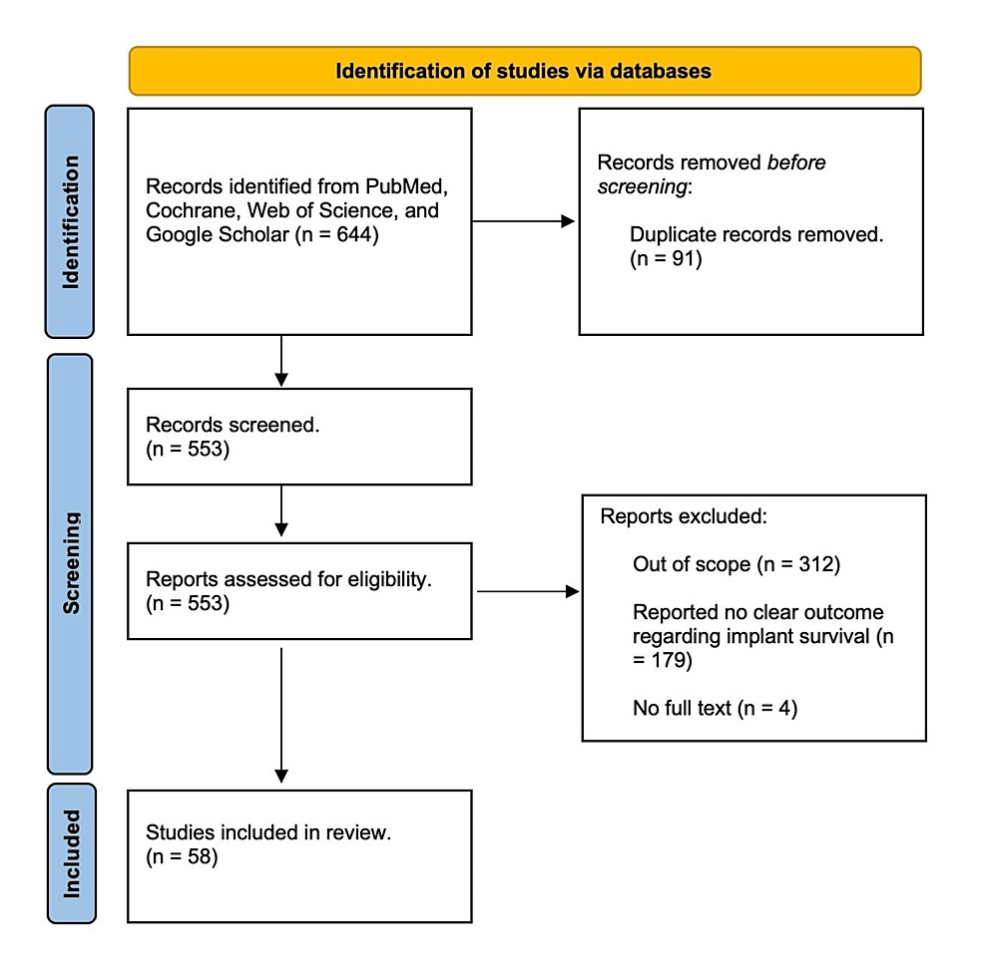
Study flow chart

In this review article, a comprehensive evaluation was conducted on published studies that discuss the utilization of iliac, fibula, and radial forearm flaps for reconstructing the mandible. Multiple databases, including PubMed, Web of Science, and Google Scholar, were utilized to gather the most pertinent literature. The search employed a variety of keywords such as “dental implants,” “free fibula flap,” “radial forearm free flap,” and “iliac flap.” Through this approach, all studies addressing the use of these flaps for mandibular reconstruction were obtained. The inclusion criteria encompassed relevant studies discussing the flaps' indications, contraindications, reconstruction methods, flap and implant survival rates, and complications. Studies with insufficient methodology, outdated information, and deficient data were excluded. Initially, 192 studies were identified through screening, but after applying the inclusion criteria, the most relevant articles (a total of 58 papers) were chosen and used in the current review. The study flow chart is presented in Figure 1.

Fibula free flap

The use of free vascularized fibula has become the preferred method for mandibular reconstruction, known as the "gold standard," since its introduction by Hidalgo in 1989. This is because it offers several advantages over other VBGs. Firstly, the fibula graft provides a substantial length of dense cortical bone, up to 25 cm in adults, along with a long pedicle based on the peroneal artery, making it suitable for reconstructing large bony defects. Over time, surgeons have adopted and improved upon this technique, resulting in multiple modifications tailored to specific individualized defect scenarios [1]. The fibula is commonly used for mandibular reconstruction and is suitable for cases requiring primarily bone and cases where the native mandible is somewhat underdeveloped. It can be used to reconstruct bony defects up to 30 cm in length, with a vascular pedicle length of 6 to 10 cm. The fibula allows for the placement of dental implants that integrate with the bone and can be easily shaped. Prior to surgery, it is recommended to evaluate the lower extremity blood vessels to assess any vascular issues that may prevent the transfer. Currently, magnetic resonance angiography has become the favored imaging technique, while research is underway to explore the potential of computed tomography (CT) angiography. Currently, there are reports on specific vascular variations found in the lower extremity. After surgery, occasional dental misalignment can occur due to improper shaping of the reconstruction plate or fractures of miniplates. Chang et al. described a successful method for treating malocclusion following fibula free flap (FFF) mandibular reconstruction by performing an osteotomy at the junction of the fibula and native mandible and realigning the mandible to achieve the correct occlusion [4]. The fibula flap is currently considered the top choice for osteocutaneous free flaps in oromandibular reconstruction. It was initially developed by Taylor in 1978 and specifically applied to oromandibular reconstruction by Hidalgo in 1989 [5].

**Figure 2:**
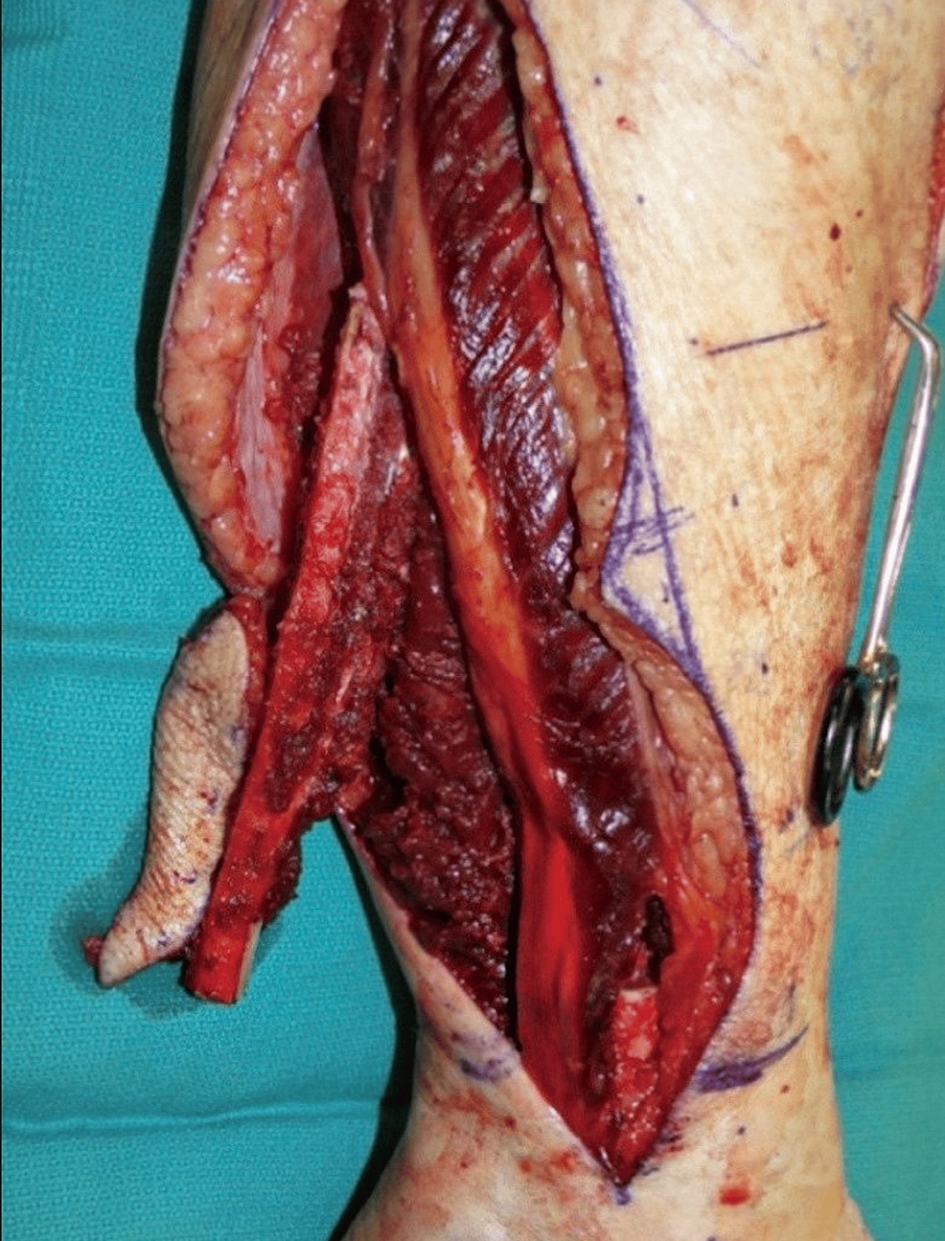
Fibula flap in situ. Image distributed under the Creative Commons Attribution Non-Commercial License [3].

After Taylor's report, the use of free vascularized fibula bone transfer became popular for reconstructing long bones [6]. The fibula has a blood supply from the peroneal artery, both through its medullary cavity and segmental periosteum, which allows it to remain vascularized even after an osteotomy as long as the periosteal system is intact. This technique was initially used to create "twin-barreled" or "double-barreled" fibula constructs to enhance load-bearing capacity in lower limb and pelvic reconstructions. Hidalgo further demonstrated that the fibula can withstand multiple osteotomies and successfully recreate the natural contours of the mandible. Early concerns about the reliability of the skin paddle, which receives blood supply from the peroneal system, have been addressed by independent investigators. The fibula osteoseptocutaneous flap offers several unique features that make it an excellent choice for reconstructing the mandible and adjacent soft tissues in most cases. These features include an abundant supply of strong, straight, and uniformly thick bone with a triangular cross-section that is long enough to reconstruct defects from one angle of the mandible to the other. The flap's structure, consisting of two layers of cortical and cancellous bone, is suitable for supporting osseointegrated dental implants. It also has a sizable and lengthy pedicle, a reliable, flexible, and sizable skin paddle that can be maneuvered to restore various intraoral and extraoral soft tissue defects, and the option to include muscle to fill dead space using a chimeric design. The flap's distal vascular runoff can be utilized for flow-through to another free flap in sequence. Additionally, the proximity of the fibula flap to the sural nerve allows harvesting of the nerve from the same donor site if reconstruction of the inferior alveolar nerve is needed. The location of the donor site allows two surgical teams to work simultaneously at the donor and recipient sites, saving operative time. Furthermore, the morbidity at the donor site is typically considered satisfactory. Different parts of the mandible experience different forces and moments. Reconstructing the anterior segment of the mandible solely with reconstruction plates has led to high failure rates due to metal fatigue and exposure. The lateral segment, although subject to less severe forces, still experiences complications such as exposure and fatigue when using plate-only reconstructions. Therefore, for some patients, a simpler and potentially palliative reconstruction may be preferable to the standard treatment. This approach is particularly suitable for elderly patients with advanced T4 cancers or for patients with low masticatory demands and short life expectancies. The free fibula osteoseptocutaneous flap is widely considered the standard treatment for mandible reconstruction in properly selected patients [6]. Bolzoni et al. explained in their study the importance of utilizing FFF in mandibular reconstruction. It offers a valuable understanding of specific aspects that require special focus in preoperative planning, patient education, and rehabilitation. Furthermore, it emphasizes the need to carefully consider the functional and aesthetic aspects of reconstruction in preoperative planning [7]. Okay et al. conducted a systematic review to illustrate the pertaining trends for utilizing the FFF during the past 10 years. The authors concluded that over the past decade, there has been a significant rise in the number of published studies focused on the utilization of the FFF for mandibular reconstruction. The number of publications in this field increased by 1.8 times, while the number of patients undergoing this procedure increased by 3.4 times in the second half of the 10-year period. Notably, the increased number of patients can be attributed to publications from the United States, India, and China. Additionally, there was a greater emphasis on computer-aided design/computer-aided manufacturing (CAD/CAM) technology in the second half of the 10-year period, which was not as prevalent in the first half [8]. In Wijbenga et al.’s systematic review, the use of implant-supported dental prostheses for oral rehabilitation after reconstructing segmental maxillofacial defects with vascularized FFF showed positive outcomes in terms of speech intelligibility and aesthetics, ranging from good to excellent. However, it is important to note that the results may be influenced by the retrospective nature of the studies. To obtain more accurate and unbiased findings, future research should focus on prospective studies that utilize standardized questionnaires and validated objective tests, with sufficient follow-up to assess functional outcomes [9]. A systematic review by Lee et al. showed that the fibula is the primary choice for reconstructive surgery in mandibular osteoradionecrosis. However, there is a lack of extensive evidence, emphasizing the necessity for larger-scale studies involving multiple institutions to identify the optimal donor tissue for the flap and assess factors that influence the success of free flap procedures. This will enable the development of personalized surgical approaches that cater to the specific needs of patients with mandibular osteoradionecrosis [10]. The fibula flap is demonstrated in Figure 2.

Reconstruction approach for fibula flap

In certain cases where there is no damage to the soft tissue and only an osseous flap is needed, the fibula flap provides versatility. The fibula graft can include skin islands, up to 25 cm long and 14 cm wide, which are suitable for reconstructing associated soft tissue defects. Multiple skin islands can be harvested with the fibula graft, based on septocutaneous or musculocutaneous peroneal perforators, allowing for the reconstruction of bony and soft tissue defects both inside and outside the oral cavity. The fibula graft has a low complication rate among osteocutaneous flaps and overcomes limitations of the extent of the skin paddle with the use of double-skin paddle FFFs. One disadvantage of this approach is the difference in height between the native mandible and the transplanted fibula, particularly at the anterior segment. This complicates the prosthetic reconstruction of the mandible. The average height of the native mandible is generally larger than the average height of the fibula, making it difficult to place dental implants for functional restoration. To address this, the technique of "double-barreling" the fibula is used. This involves osteotomies and folding over the fibula graft to create equal struts while preserving the blood supply throughout the graft. This technique allows the double-barreled fibula flap to match the height of the mandible, resulting in better aesthetic and functional outcomes. It also enables a one-stage procedure with immediate osseointegrated dental implantation. While the vertical distraction technique has been used to expand the neomandible for delayed dental implantation, recent data suggest increased complexity and complications compared to double-barreled fibula grafts. Therefore, double-barreled fibula grafts are recommended for osseointegrated dental implants. Depending on the purpose of reconstruction, the fibula can also be partially double-barreled. This technique has shown high flap survival rates and good aesthetic and functional outcomes [3]. It is recommended to always raise the fibula with a skin paddle. When soft tissue restoration is not necessary, the skin paddle serves as an effective way to monitor the vascularity of the bone, eliminating the need for implantable monitoring devices. The peroneal septocutaneous vessels, which supply blood to the skin, are located at the posterior edge of the fibula and are concentrated in the middle and distal thirds of the leg. Preoperatively, these vessels can be marked using a pencil Doppler probe. Depending on the number and size of the septocutaneous vessels, a skin paddle area of up to 14 x 25 cm can be reliably based on one or two vessels. Retrograde dissection of myocutaneous perforators is rarely needed unless the planned skin paddle lacks sufficient blood supply, in which case the perforators may be sacrificed to facilitate flap harvesting and increase paddle mobility. Additionally, if more tissue bulk is required, a segment of the soleus muscle can be included, but the potential impact on donor site morbidity should be considered. To enhance the rotational mobility of the skin paddle, the posterior crural septum can be divided on both sides of the selected septocutaneous vessels during insetting [6]. On the day of the surgery, guides are used to assist with both the resection and fibula harvest. For the mandible, a cutting guide with corresponding holes to the reconstruction plate is used. These holes are drilled prior to making the cuts, ensuring accurate placement of the bone segments using the plate as a guide. Tooth-borne or bone-borne cutting guides can be used, with tooth-borne guides offering precise indexing if enough stable teeth are present. Placing the fibula cutting jig precisely on the leg and securing it with screws prevents movement. Accurate osteotomies are crucial for the final reconstructive plan. Dental implants should be placed in the fibula before completing the closing osteotomies to ensure a long linear bone segment that does not rotate during implant delivery. Implants are placed in a flapless technique to prevent damage to the fibula flap's blood supply. Careful dissection of muscle and perforating vessels away from the implant sites is necessary. Over-torqueing implants can lead to stress risers and potential fractures or bone loss. Muscle fibers from the fibula flap interfering with implant placement should be gently dissected. Screw-tapping the osteotomies is often required for complete insertion of the implants. Once placed, the implants are hand-torqued to the appropriate value. The implants are generally placed at the bone level or slightly supracrestal due to the sloped surface of the fibula, and it is not uncommon for a thread or two of the implant body to be exposed on the buccal side. Prosthetic abutments with screw-retained temporary cylinders are placed and torqued, and the fibula osteotomies are completed. The provisional prosthesis is attached using premilled locator holes, acting as an external fixator for the fibula segments. The reconstruction plate is adapted to the fibula flap using predrilled holes, and the entire construct is tried into a model of the defect site. Adjustments are made to ensure proper occlusion before the provisional prosthesis is cemented to the temporary cylinders. The completed construct is then transferred to the face and placed into maxillomandibular fixation precisely aligned with the opposing arch. Small adjustments may be necessary during the bone graft alignment process to correct any misalignments or interferences between the bone and plate. Recognizing and addressing these issues is crucial for a successful outcome [11]. Lin et al. illustrated that the use of a single fibular transfer can address two jaw bone defects and reconstruct the facial profile. It can improve oral functionality by resolving oral contracture and enhance the aesthetic appearance by correcting sunken cheeks. Additionally, the reconstructed fibula can enable rehabilitation through the implantation of secondary teeth [12]. Moreover, Bolzoni et al. explained that the combination of various microinvasive methods and the conventional approach can be seamlessly interchanged and safely used together when the underlying defects permit it [13]. In many European maxillofacial surgical units over the past decade, there have been recognized "tricks" for the fibular free flap procedure. These techniques include several steps. Firstly, when the recipient vessels are far apart, the distal fibula is harvested. Secondly, the selection of the flap is based on the anatomy of the perforators. Thirdly, the flap's skin paddle is utilized for monitoring purposes after the operation. Fourthly, it is crucial to protect the soft tissue cuff of the flap. Fifthly, preventing venous thrombosis is essential to minimize complications related to the flap. Moreover, when employing the double-barrel technique, it is important to align the fibular struts and safeguard the vascular pedicle. Additionally, reducing the gap between the double-barrel struts and ensuring long-term follow-up of dental implants is necessary. Proper selection of osteosynthesis materials is an important consideration as well. Lastly, it is important to take into account the learning curve and clinical competence in microvascular reconstruction [14]. Efforts to enhance occlusal accuracy and operational efficiency in mandibular reconstruction are being augmented through the utilization of cutting templates, three-dimensional (3D) printing models, and prebent plates [15]. The utilization of modern virtual technology and equipment facilitates the precise positioning of guided implants and fibulas in restorative locations, enabling immediate dental rehabilitation [16].

Implant placement considerations with fibula free flap

To achieve reconstruction goals, which include maintaining integrity, optimizing function, restoring form, minimizing complications, and enhancing quality of life, several factors must be considered for patients needing free tissue transfer and rehabilitation with an implant-supported prosthesis. Key factors include patient motivation, survival, and long-term prognosis. It is important to outline the individual's specific reconstruction goals, manage patient expectations, ensure the availability of a reconstructive team, and facilitate effective communication among the patient, reconstructive surgeon, and prosthodontist. The prosthodontist should possess expertise in rehabilitating compromised surgical sites and be committed to seeing the patient through treatment and follow-up for prosthesis maintenance and hygiene. Anatomic factors, such as suitable tissue harvest and microvascular anastomosis, maxillomandibular relationship, bone volume and location, remaining dentition, mouth opening, and the function of the tongue, lips, and pharyngeal wall, play a critical role in patient evaluation for reconstruction. Patient-related factors, including comorbidities that may prohibit free tissue transfer or a history of head and neck irradiation, should also be taken into account. It is necessary to discuss the timing of surgeries, rehabilitation steps, the importance of meticulous oral hygiene, and the cost of implant placement and rehabilitation before initiating treatment [2]. Generally, endosseous implants can be placed in FFF either immediately at the time of fibula harvest or be delayed by 6-12 months. Both primary and secondary implant placement methods have similar rates of implant survival, complications, and safety. Alternatively, reconstruction can be performed using prefabricated flaps or a single-stage complete maxillofacial reconstruction known as "jaw in a day," each with its own advantages and disadvantages. Primary implant placement in FFF is considered safe and does not compromise the vascular supply to the fibula bone. This procedure improves functional outcomes and reduces overall reconstruction time by minimizing the number of procedures. Immediate implant placement offers benefits such as easier access to the fibula bone, fewer surgeries, lower costs, early oral rehabilitation, faster return to oral nutrition, and prosthesis use, and allows time for implants to integrate before radiation therapy, potentially reducing the risk of implant loss and osteoradionecrosis. However, there are disadvantages such as improper positioning of implants, potential interference with radiation therapy, alteration of local anatomy, and the possibility of tumor recurrence at the implant site, making primary implant placement contraindicated after malignant tumor removal. Secondary implant placement is a two-stage technique. In the first stage, the defect is reconstructed with an FFF and reconstruction plate, allowing for four to six months of healing. In the second stage, CAD/CAM technology is used to guide the surgeon in optimal implant placement. For patients with malignant tumor defects treated with FFF and radiation therapy, the secondary placement of implants is typically delayed for one year after completing therapy. Reconstructive hardware is removed if it interferes with implant placement. Additional procedures such as debulking of the flap skin paddle, vestibuloplasty, and grafting can be performed during implant placement or uncovering. Secondary implant placement offers advantages such as identifying motivated patients for further prosthetic rehabilitation, shorter initial surgical procedures, confirmation of the viability of FFF, and creation of an ideal implant placement guide and prosthodontic plan, and allows time to rule out local tumor recurrence. However, disadvantages include delays in oral rehabilitation, more trips to the operating room, and increased costs [2]. In primary reconstruction, implants are placed simultaneously with the FFF reconstruction. This method is well-established, safe, and effective. It is suggested that the negative effects of radiation therapy do not occur until six weeks after initiation, allowing sufficient time for the implants to integrate with the bone. For secondary reconstruction, implant placement is delayed for four to six months to allow for the healing of osteotomies and integration of soft tissues before starting oral prosthetic reconstruction. Waiting for more than six months does not impact implant survival, as reported by Curi et al. in 2018. Generally, the minimum waiting period after completing radiation therapy is six months, but waiting for 12 months is ideal to reduce the risk of implant failure. The implant brand does not affect implant survival, as most commercially available systems are compatible with FFF restoration. Implants with a treated surface have fewer failures compared to machined implants, likely due to the larger surface area and faster osseointegration. Short implants have higher failure rates in irradiated areas compared to standard-size implants (10 mm or longer). Based on these findings and the authors' experience, a surface-treated implant with a width of at least 3.5 mm and a length of 10 mm should be used for FFF reconstruction [2]. Aesthetic and functional reconstruction of the mandible can often be achieved by combining the transfer of free tissue with the placement of implants that integrate with the bone to support dental prostheses. Implant placement requires a bone height of approximately 6 to 7 mm. Implants placed and loaded in reconstructed bone perform similarly to those placed in native bone. Secondary placement of osseointegrated implants after mandibular reconstruction is a safe and reliable technique for oral rehabilitation. There are also successful reports of primary implant placement during mandibular reconstruction. However, in cases where postoperative radiation is planned, implant loading is delayed for at least six months. Chang et al. in 2003 described a technique for accurately placing implants during primary insertion in conjunction with vascularized free fibula transfer. They recommend using waxing screws connected to the implant fixture to assess implant topography, ensuring accurate determination of the direction of occlusal loading force and preventing tilting of the fibula-implant construct during fixation to the reconstruction plate. Leung et al. in 2003 conducted a study on functional and quality-of-life outcomes of dental implants in reconstructed jaws. They found that the majority of patients (85%) reported satisfaction with their treatment outcome and had no issues with daily activities, including dining in public [4]. There are challenges in oral rehabilitation using dental implants that integrate with the bone. Firstly, the existing bone asymmetry leads to an unfavorable relationship between the implant and crown. Additionally, the success rates of dental implants placed in grafted bone are lower compared to non-grafted bone. Furthermore, radiotherapy further decreases the success rates of dental implants [1]. Primary implantation is beneficial in reducing the time needed for rehabilitation, particularly in cancer patients, and it also has a success rate comparable to that of secondary implants. However, if delayed implant placement is preferred, it should be done at least six to 12 months after the graft, once muscle healing and bone remodeling have been completed, to avoid implant failure caused by improper positioning [17]. Gangwani et al.’s systematic review and meta-analysis investigated how successful osseointegrated dental implants are when placed as a second step in FFFs, using the criteria developed by Albrektsson and colleagues. The findings of this thorough review and analysis suggest that employing objective standards, the placement of dental implants in FFFs with a delay is remarkably effective [18].

Complications associated with fibula flap

Donor sites are consistently linked to significant morbidity. For instance, a study revealed that 24% of patients who underwent FFF reconstruction for the mandible experienced ongoing walking difficulties even after eight years, while 34% were unable to run during the same period. The study also found that 21% of patients required regular pain medication. Moreover, there was a decrease in foot movement range, extension, and flexion strength, as well as decreased sensitivity at the donor site [1]. The primary disadvantage of using the fibula for mandible reconstruction is its relatively small height, which is approximately 13 mm. This poses a challenge when addressing defects in the anterior segment because there is a trade-off between restoring sufficient alveolar height for dental implants and achieving the desired facial height with an aesthetically pleasing inferior mandibular margin. Placing the fibula at the inferior mandibular margin results in a good contour, but it requires elongated prosthetic suprastructures for occlusion of osseointegrated implants in this position. This can lead to excessive lever arm forces that pose a long-term risk to the success of the implants. We have identified three methods that are useful in addressing this issue [6]. The common early complications in the healing of donor areas include delayed healing, bleeding, and infection. However, these complications typically do not lead to long-term issues and do not impact the functional outcome [19]. Microvascular osteocutaneous free flaps have provided reconstructive surgeons with an effective method to reconstruct complex defects in head and neck surgery. Flaps such as the radial forearm, scapula, iliac crest, and fibula have been widely utilized in mandibular reconstruction. However, there has been less focus on the potential negative impacts on the donor site as compared to the numerous reconstructive benefits offered by these osteocutaneous flaps [20].

Radial forearm free flap

The radial forearm free flap, first described in 1981 [21,22], provides an opportunity to address facial defects with its long vascular pedicle and elasticity. However, it lacks the necessary rigidity for nasal framework reconstruction [23]. The radial forearm free flap has become the preferred choice for oral cavity soft tissue reconstructions due to its versatility and ability to reconstruct large- and medium-sized defects [24]. It is commonly used for head and neck defect restoration, including full-thickness lip and oral cavity defects [22]. One drawback is the noticeable forearm scar that remains after the procedure. Although the defect in the skin is typically treated with full-thickness grafting or split-thickness grafting, there is a potential for severe morbidity at the donor site and poor aesthetic outcome, as well as restrictions in forearm function and healing issues [25].

While the radial forearm free flap provides a sensate, pliable, and thin skin paddle with a long vascular pedicle and easy technical harvest, it can lead to severe donor site morbidity, particularly from flexor tendon exposure resulting from unsuccessful skin graft harvesting, as well as altered sensation in the radial nerve, aesthetic deformity, reduced range of motion, and grip strength [22]. The lateral arm flap offers a distinctive and highly versatile soft tissue flap, allowing continuous alterations with exceptional contour results in different sites and forms [26]. By utilizing a modified suprafascial technique, the risk of flexor tendon exposure can be reduced by preserving the deep area of the fascia overlaying the tendons, resulting in an improved graft recipient bed [22].

When a rigid support system is required, especially in cases of postoperative radiation or large bony defects of the skull base without alternative options, the use of an osteocutaneous radial forearm free flap is indicated. However, there are several considerations regarding this procedure that should be acknowledged [27]. It is recommended that this procedure be performed by a multidisciplinary team, as the radial forearm free flap is associated with common postoperative complications and significant morbidity [27].

To address the disadvantages of the radial forearm free flap, the ulnar forearm fasciocutaneous flap can be used as an alternative. Introduced in 1984 by Lovie et al. [22], it offers similar advantages to the radial flap but with less donor site morbidity and superior tissue characteristics. The sensation of the ulnar nerve is typically unaffected, the skin of the flap is less hairy than that of the regular radial flap, and fewer donor site defects are observed due to the location of the skin paddle. However, the ulnar forearm flap is seldom used due to the perception that harvesting these flaps is challenging and the belief that the ulnar artery is crucial for hand circulation [22].

**Figure 3:**
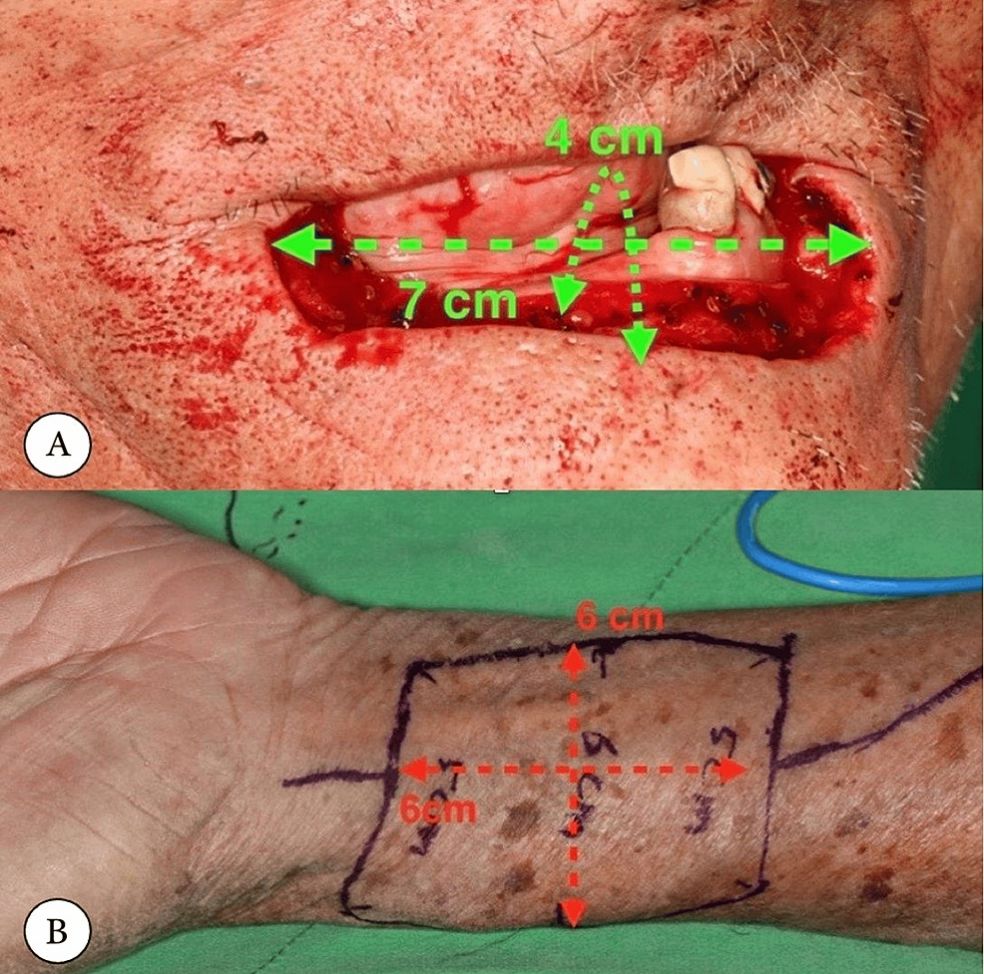
(A) The defect spans 4 cm vertically and 7 cm horizontally. (B) The free forearm flap was acquired measuring 6 × 6 cm. Image distributed under the Creative Commons Attribution Non-Commercial License [28].

The Allen test is frequently employed to assess the contributions of the ulnar and radial arteries to palmar arch perfusion [22]. However, this test is considered unreliable and subjective, with a sensitivity of 91.7% and a specificity of 54.5% [22]. To address the limitations of the Allen test, many surgeons now use various techniques, such as intraoperative testing and pulse oximetry, to improve its accuracy [22]. It is worth noting that the radial artery carries the primary vascular supply to the hand, and previous research has shown that the radial artery is smaller than the ulnar artery in the proximal forearm but has a larger diameter at the wrist [22]. The lateral arm flap has been introduced in the literature as an alternative to the traditional radial forearm flap, primarily due to improvements in the characteristics of the flap [26]. A demonstration of the radial forearm free flap and flap size is shown in Figure 3.

Reconstruction approach for radial forearm free flap

While the radial forearm free flap has certain advantages, it also presents with several issues [29]. Skilled surgeons with advanced experience and training in free tissue transfer are required for this procedure [29]. Additionally, specific equipment such as microsurgical instruments and an operating microscope are necessary, and the procedure itself is often lengthy and time-consuming [29]. The blood supply to the forearm is somewhat variable and relies mainly on the brachial artery, which divides into the radial and ulnar arteries [23]. The ulnar artery, located superficial to the flexor retinaculum, contributes to the superficial palmar arch when entering the hand [23].

A new method known as the shape-modified technique has emerged for the radial forearm free flap [25]. This approach allows for the primary closure of the defect, eliminating the need for skin grafts [25]. It involves designing narrow flap strips based on the cutaneous perforators that traverse the radial artery pathway [25]. These strips can be divided and folded as necessary to match the defect and achieve the desired width [25].

In one study, Balakrishnan et al. evaluated the effectiveness and outcomes of a single-stage reconstruction technique using a combination of the Pacman-style free radial forearm flap and oblique elastic musculomucosal flap for composite post-excisional commissure and pericommissural defects [30]. The author suggested that this combination of flaps in single-stage reconstruction is a valuable adjunct in restoring continence and aesthetics at the reconstructed site [30]. On the other hand, Piotrowska-Seweryn et al. presented a combined flap of radial forearm free flap and auricular free flap for nasal defect reconstruction [23]. The author reported satisfactory aesthetic outcomes and concluded that this flap combination appeared to be optimal for extended nasal defects [23]. The use of a double skin paddle radial forearm free flap in the management of tracheoesophageal fistula was described in the literature, which enables the rebuilding of both the tracheal and pharyngoesophageal walls, while also inserting protective tissue between the two suture lines [31]. Moreover, Papanikolas et al. explained that each type of flap serves a crucial purpose in repairing oral defects, with anterolateral thigh flaps being the preferred choice for larger defects. However, for smaller defects, the superficial circumflex iliac artery perforator (SCIP) flap is a dependable alternative that yields outstanding results before and after surgery [32]. De Santis et al. explained that the prelaminated fasciomucosal radial forearm free flap is a viable choice for reconstructing the tip of the tongue, particularly in specific cases that prioritize clear speech and proper articulation [33]. Reconstructing a complete lip defect and restoring the vermilion border of the lip presents a difficult task [34]. Rahman et al. conducted a study on total lip reconstruction involving five patients at Sylhet MAG Osmani Medical College Hospital and a private clinic in Sylhet, Bangladesh, between January 2014 and December 2017. One patient had basal cell carcinoma of the upper lip, while the others had squamous cell carcinoma of the lower lip. The average age of the patients was 71 years. All the flaps used in the procedure were successful, resulting in normal speech and oral control. The composite radial forearm flap with palmaris longus tendon proved to be a dependable and effective option for total lip reconstruction [35]. Maruccia et al. reported a case involving a 45-year-old woman who presented with a history of palate squamous cell carcinoma and a severe nasal deformity resulting in almost complete loss of the columella. To address this issue, a two-staged reconstruction using a prelaminated radial forearm free flap with the fifth rib was performed. The radial pedicle of the flap was connected to the facial vessels, and the patient experienced a smooth postoperative recovery. The flap survived completely, and after 10 months, the patient had normal breathing through both nostrils, an improved projection of the nasal tip, and a satisfactory aesthetic outcome. The study suggests that a prelaminated radial forearm free flap can be a valuable option for reconstructing a large composite defect of the columella when adjacent tissues are limited [36].

Implant placement considerations with radial forearm free flap

The strategic coordination of implant placement along with RFFF is crucial for optimizing patient outcomes. It is important to carefully assess factors such as the timing of implant placement, the survival rate of the implant, and the trajectory of wound healing. The specific timing of implant placement depends on individual patient characteristics and case details. However, it is generally agreed upon that allowing sufficient recovery time before implant intervention can improve treatment success [37].

When integrating a radial forearm free flap with an implant, there are various considerations. These include ensuring proper vascularization to the flap, precise positioning and stabilization of the implant, and ensuring adequate coverage of the implant by the flap. In a particular study, a patient-specific polyether ether ketone (PEEK) implant was used for reconstructing the zygomatic arch and was secured with a plating system. The survival probability of the implant and considerations related to wound healing largely depend on the patient's health status, postoperative care, and the potential for complications following surgery. In this specific case, the combined use of a PEEK implant and a radial forearm free flap was successful [38].

The implant's survival rate, when combined with the endoscopic adipofascial radial forearm free flap reconstruction approach, was found to be 100%, indicating full viability of the flap. The positioning of the implant takes place after endoscopic resection and careful evaluation of the defect size. The wound healing rate was also notable, with 83% of patients showing successful wound healing and effective separation between the sinonasal cavity and the intracranial space [39].

Complications of radial forearm free flap

The radial forearm free flap is associated with various adverse complications such as thenar hypoesthesia, wound infection, and hematoma [29]. Many of these flaps require coverage of the donor site with a split-thickness graft, which can lead to further complications such as tendon exposure and skin graft loss, resulting in wrist dysfunction [29]. Mai et al. conducted a study to assess the donor site morbidity of the radial forearm free flap and posterior tibial artery perforator flap following cancer removal [40]. The study findings indicated no significant difference in wrist and ankle movement ranges before and after surgery [40]. Additionally, Kim et al. proposed a strip design that simplifies the creation of an externalized monitoring flap using a buried radial forearm free flap without additional donor site morbidity [41]. Flap shrinkage is a commonly observed effect during follow-up after flap transplantation and radiotherapy. This shrinkage can influence both aesthetic and functional outcomes. To mitigate this effect, some authors advocate for oversizing compound flaps in reconstruction following craniomaxillofacial tumor resection [42].

Ibrahim et al. compared the outcomes of using radial forearm free flaps and facial artery musculomucosal flaps for the reconstruction of medium-sized oral cavity defects, with a focus on functional and surgical outcomes [24]. The study demonstrated that the facial artery musculomucosal flap can be employed to reconstruct medium-sized defects with similar functional outcomes as radial forearm free flaps while reducing morbidity and costs associated with the procedure [24]. In a rare case reported by Bitner et al., a head and neck chondrosarcoma originating from the mandibular coronoid process was reconstructed using a custom PEEK implant, with contour restoration achieved using a radial forearm free flap [38]. Ranganath et al. conducted a study comparing aesthetics, function, surgical morbidity, and health-related quality of life in patients who underwent intraoral reconstruction using either a radial forearm free flap or an anterolateral thigh free flap [43].

A study demonstrated that the anterolateral thigh free flap yielded lower donor site morbidity, comparable oral function, and survival rates when compared to the radial forearm free flap [43]. Gschossmann et al. conducted a study discussing the success rates and feasibility of contralateral anastomosis in free flap reconstruction [21]. The study concluded that contralateral anastomosis is a safe and effective option for reconstructing head and neck defects, particularly when using a radial forearm free flap [21]. Krane et al. compared the aesthetic outcomes and morbidity of split-thickness skin grafts and full-thickness skin grafts for reconstructing the donor site of the forearm free flap [44]. The author suggested that employing full-thickness skin grafts for the donor site reconstruction of the forearm free flap provides superior aesthetic outcomes without the morbidity associated with split-thickness skin grafts [44]. In a study by Gur et al., different free flap options for intraoral lining and tongue reconstruction were compared, with the author recommending the anterolateral thigh flap as the most suitable choice for average-weight individuals due to its pliability, reliability, and consistent vascular structure [45].

Iliac flap

The mandible, or lower jaw, is crucial for both facial aesthetics and functional abilities. Vascularized free bone grafts have become the favored method for mandible reconstruction. These grafts have high success rates and focus on optimizing function and aesthetics. Various free flap options have led to significant advancements in mandibular defect reconstruction, resulting in improved functional outcomes and aesthetic results. The mandible plays a vital role in essential functions such as occlusion, mastication, swallowing, speech, and maintaining oral competence. Defects in the mandible can result from cancer surgeries, trauma, infections, or osteoradionecrosis. Reconstructing these defects is challenging for surgeons specializing in head and neck procedures. Experienced microvascular surgeons have reported success rates above 90%, demonstrating their proficiency in these procedures [46]. Free flaps, particularly osteocutaneous flaps, provide reliable soft tissue coverage and bony reconstruction for the mandible. These flaps include both skin and soft tissue along with bone, allowing for optimal reconstruction of complex defects. Vascularized iliac bone grafts have been used since 1979, and modifications have been suggested to enhance reliability, reconstruct three-dimensional defects, and improve overall outcomes [47]. Free vascularized osteocutaneous flaps offer several advantages over conventional bone grafts. They promote healing through callus formation, have attached vascularity for primary healing, reduce complications, and can survive in compromised recipient beds. These flaps can be transferred in a single stage, allowing patients to resume their regular activities sooner. They are particularly beneficial for older patients, preserving their quality of life. In younger patients, these flaps provide reliability and support for dental implants [48]. The advancement of the iliac crest microsurgical free flap has significantly improved the surgical reconstruction of the mandible [49]. The iliac crest is a preferred choice as a donor site for mandibular reconstruction. It closely matches the shape and curvature of the mandible, facilitating anatomical contouring. The iliac crest flap provides ample bone supply and includes sufficient skin and soft tissue for reconstructing composite defects. Although the muscle portion of the flap may appear bulky, it can be utilized for closing soft tissue defects through secondary epithelialization. Overall, the use of the iliac crest as a donor site for mandibular reconstruction offers anatomical compatibility, ample bone supply, and versatile soft tissue components, making it a valuable option for addressing complex mandibular defects [46].

When dealing with non-aligned skin and bone defects in different planes, various approaches can be employed for mandibular reconstruction. One method involves using larger skin flaps while ensuring an adequate supply of the bone. Another alternative is obtaining a vascularized bone segment that closely matches the contour and structure of the mandible, such as an iliac bone graft with ample height and thickness. This provides an ideal substrate for seamless integration with osteointegrated implants. To reduce the bulk of the flap, the internal oblique abdominis muscle can be used as the primary component of the myofascial flap. By attaching the internal oblique muscle to the iliac crest, the size and volume of the flap can be significantly decreased. While some researchers have suggested that the vascularized internal oblique-iliac crest myoosseous free flap is an optimal choice for restoring oral mucosa defects within the oral cavity, it is important to note that preserving the ascending branch of the deep circumflex iliac artery (DCIA) and vein within the flap can be challenging, particularly for less experienced surgeons. Careful surgical skills and experience are required to ensure the successful preservation of vascular structures within the flap. The soft tissue component of the iliac crest flap receives a reliable and stable blood supply from the nourishing vessels of the iliac crest. This vascular supply ensures adequate perfusion to the soft tissue, promoting healing and overall flap viability. Additionally, using the iliac crest flap offers the advantage of a shortened healing period. This is due to the direct suturing of the fascia with the recipient mucosa, which facilitates a more efficient and accelerated healing process. The direct connection between the fascia and mucosa allows for improved integration, faster tissue regeneration, and wound closure [50].

When dealing with a soft tissue defect in conjunction with mandibulectomy, the use of the skin paddle from the iliac crest flap can present challenges. The skin paddle of the iliac crest flap often leads to excessive bulk and thickness in the reconstructed area, resulting in suboptimal therapeutic outcomes. These outcomes can include compromised aesthetics and functional limitations. Therefore, alternative options such as the fibula flap or other flap techniques may be more suitable for addressing soft tissue defects, offering more favorable outcomes in terms of thickness and overall appearance [50]. The SCIP flap is commonly utilized in areas with relatively flat surfaces, such as the extremities. However, it may not be the most preferable option for regions characterized by numerous folds, such as the intraoral region [51]. Qiu et al. conducted a study to assess the precision of mandibular reconstruction using the iliac flap, guided by a set of digital surgical guides. Seven patients were included in the assessment of post-surgical reconstruction accuracy. These patients underwent mandibular reconstruction using a vascularized iliac flap, with the guidance of digital surgical guides. The authors concluded that this approach to mandibular reconstruction, utilizing the iliac flap and digital surgical guides, is both accurate and effective [52]. The objective of Zho et al.'s study was to compare the accuracy of mandibular reconstruction using complicated guiding templates (CGTs) versus simple guiding templates (SGTs) and to determine whether the additional time spent designing complicated templates is necessary before surgery. The study found that in cases of straightforward mandibular reconstruction, the CGT procedure, which takes more time, did not significantly impact symmetry and midline displacement compared to the SGT procedure. However, it did lead to less reduction (thus greater preservation) in alveolar height and a smaller gap where the bones join together. Additionally, CGTs reduced average operation time and simplified intraoperative procedures compared to SGTs [53]. The extensive utilization of DCIA flaps in oromandibular reconstructions has been hindered due to the excessive size of the "obligatory muscle cuff" and the attachment of the skin to the bone, which makes the placement of soft tissue challenging, despite the many benefits of using the iliac crest [54].

Implant placement considerations with iliac flap

The use of the iliac crest flap provides sufficient bone height, which is essential for effective plate fixation and the placement of dental implants during dental restoration. This flap offers a large bone volume, favorable shape, and ample height, making it an ideal choice for some surgeons when it comes to plate fixation and implant placement. It reliably reconstructs mandibular defects while enabling successful implant integration, leading to optimal functional and aesthetic outcomes [55]. Compared to a fibular flap, the iliac flap is more suitable for implant placement and reconstruction based on occlusion [56]. During the procedure, the iliac crest was raised while retaining its cortex. Afterward, it was moved to the defect in the mandible, positioned in an outward direction, and acted as a foundation for the alveolar process. This technique enhances implant stability and minimizes bone resorption, resulting in improved long-term outcomes. Three-dimensional CT photographs were used to effectively illustrate the aesthetic and functional outcome of the final mandibular reconstruction since the axial images from the CT scan did not show a significant correlation. The aesthetic outcome of the mandibular reconstruction using the iliac crest flap is highly pleasing, largely due to the remarkable similarity in structure between the iliac crest and the mandibular body. The integrity of the mandible's inferior border is well-preserved, leading to the retention of a favorable occlusal relationship. The myofascial component of the iliac crest flap undergoes a transformation process, gradually acquiring tissue characteristics similar to the oral mucosa. Within a week after the surgery, the exposed myofascial component exhibits an irregular and necrosis-like appearance, indicating the initiation of secondary epithelialization. The absence of a skin flap facilitates accelerated healing and early integration of the fascia with the adjacent oral mucosa, often observable within as little as 10 days postoperatively. Over time, it becomes challenging to distinguish between the reconstructed mouth floor and the oral mucosa in the recipient area, as their appearance becomes nearly indistinguishable within approximately one month. The myofascial tissue of the flap exhibits a smooth and moist surface, closely resembling the texture of the adjacent oral mucosa. The use of the myofascial component of the iliac crest flap yields notable improvements in both aesthetic and functional aspects. Its superior color match and texture surpass outcomes achieved with conventional skin flaps, resulting in enhanced overall results for the patient [50]. According to several authors, the success rate of placing dental implants in vascularized iliac crest grafts is remarkably high, with some studies indicating an impressive 95.2% success rate [54].

Complications of iliac flap

Femoral Nerve Palsy

This complication was associated with anticoagulant therapy during the perioperative period rather than the iliac flap itself. Physical therapy was provided, leading to significant improvement and near-complete neurological function in the femoral nerve within six months.

Arterial Spasm and Flap Failure

Arterial spasm caused the failure of the iliac flap in some cases. A successful free iliac bone transplantation was performed to address this issue, resulting in a high survival rate of the bone graft (95.6%). Minor skin-edge necrosis occurred in a few patients but resolved with appropriate dressing changes. The reconstructed mandible achieved the desired anatomical contour with satisfactory length and vertical height.

Donor Site Complications

No significant complications were observed at the donor site throughout the follow-up period. Some patients reported mild pain at the donor site, but there were no hernias, bone fractures, or gait disturbances.

Sensory Deficits

Some patients experienced sensory deficits along the lateral femoral cutaneous nerve distribution postoperatively, but symptoms gradually improved over time.

Infection During Postoperative Chemoradiotherapy

A patient with oral carcinoma encountered a local infection during postoperative chemoradiotherapy, but it was successfully treated without causing delays in adjuvant therapy.

Abdominal Subcutaneous Fat in Female Patients

Increased abdominal subcutaneous fat was observed in some female patients, which resolved over a period of two to three months during follow-up, resulting in thinner and more suitable skin flaps [46].

Myofascial Tissue Complications

In one case, granulation-like and fibrotic tissue developed on the surface of the myofascial tissue within two weeks postoperatively. A revision surgery was performed using a biological membrane to promote proper healing [50].

Orocutaneous Fistula

In three patients, a purulent orocutaneous fistula was observed but managed successfully without additional surgical intervention, using conservative measures such as antibiotics and proper wound care.

Flap Necrosis

In one patient, necrosis of both free flaps (iliac crest flap and fibula flap) occurred. This is a significant complication that can compromise the success of the surgery. The patient had a history of recurrent ameloblastoma, which may have influenced tissue condition and affected the outcome of the flap reconstruction.

Superficial Circumflex Iliac Artery Perforator Flap Failure

Perforator and perfusion issues led to the failure of SCIP flaps, with one flap experiencing partial necrosis. Flap thickness tends to be increased in patients with obesity. It is important to note that these outcomes are based on specific cases and individual variations may occur. Each patient's condition and response to treatment should be evaluated on a case-by-case basis to establish the most appropriate management and treatment options [45].

Comparison between each flap

In general, FFF and iliac crest flap have similar outcomes in terms of donor site morbidity, pain scores, walking ability, wound healing, speech, oral competence, aesthetics, and quality of life. They also provide comparable bone volumes for implant placement, minimal bone resorption (<1 mm), and stability during the uncovering process. However, FFF reconstruction tends to result in less gait and neurosensory changes in the extremities compared to iliac crest flap reconstruction. Complications such as seroma, hematoma, and herniation are more limited to the iliac crest flap donor site. Swallowing is generally better in patients reconstructed with FFF, likely due to the larger skin paddle of iliac crest flap. The bulky intraoral skin paddle of the iliac crest flap may require significant reduction for proper emergence profile and implant hygiene. Iliac crest flap is typically the first choice for dentate patients with mandibular angle and body defects that require a bulkier soft tissue skin paddle. FFF reconstruction is preferred for edentulous patients or dentate patients with longer-span defects (subtotal or total mandibulectomy) and those requiring condylar reconstruction. While the deep circumflex iliac artery offers a greater vertical bone dimension, considering the above data, FFF is generally a better option for maxillofacial reconstruction in most patients, with iliac crest flap serving as a reliable alternative for specific cases [2]. The selection of an appropriate free flap should be tailored to the size and location of the tumor, as well as the specific needs of each patient. Advanced techniques, such as virtual surgical planning using 3D models and intraoperative cone-beam CT, can enhance the precision of the reconstruction, leading to better outcomes and improved quality of life for the patient [56].

A previous meta-analysis by Lonie et al showed that when considering mandibular reconstruction, both iliac flaps and FFFs are viable options. The choice between the two depends on factors such as the patient's pre-existing condition (age, mobility, size, healing ability), the size and location of the defect, and the intended reconstruction plan (including the use of dental implants). For mandibular angle or body defects that require contour and soft tissue bulk reconstruction or dead space-filling, the iliac crest flap is recommended due to its advantageous contour. On the other hand, if bony length is needed, particularly for subtotal or total mandibulectomy, the fibula flap is the more suitable choice [57]. Haughey et al. described the possibility of using free, non-bulky muscle flaps for reconstructing mucosalized oral soft tissue in specific patients. This technique involves utilizing the fibular donor site as the primary option and the iliac donor site as the secondary option. Additionally, this soft tissue approach also helps with prosthetic dental rehabilitation [58].

Limitations

This comprehensive review article focused on the survival of dental implants placed in fibula, radial, and iliac flaps. More studies can be conducted to investigate the long-term survival of these dental implants.

## Conclusions

This review illustrates that dental implants placed in fibula, radial, and iliac flaps demonstrate a high survival rate. However, some points must be considered by the surgeon. The common early complications in the healing of donor areas include delayed healing, bleeding, and infection. However, these complications typically do not lead to long-term issues and do not impact the functional outcome. The decision between which flap to use depends on several factors, including the patient's age, mobility, size, and healing ability, as well as the size and location of the defect and the planned reconstruction, including the use of dental implants. This review explains that the iliac crest flap is recommended due to its advantageous contour for mandibular angle or body defects requiring contour and reconstruction of soft tissue mass or filling dead space. However, if there is a need for longer bone length, especially in cases of subtotal or total mandibulectomy, the fibula flap is a more suitable choice.
